# Beneficial Effect of Bariatric Surgery on Abnormal MMP-9 and AMPK Activities: Potential Markers of Obesity-Related CV Risk

**DOI:** 10.3389/fphys.2019.00553

**Published:** 2019-05-08

**Authors:** Concha F. García-Prieto, Marta Gil-Ortega, Elena Vega-Martín, David Ramiro-Cortijo, Miriam Martín-Ramos, Elena Bordiú, Andrés Sanchez-Pernaute, Antonio Torres, I. Aránguez, María Fernández-Alfonso, Miguel A. Rubio, Beatriz Somoza

**Affiliations:** ^1^Departamento de Ciencias Farmacéuticas y de la Salud, Facultad de Farmacia, Universidad San Pablo-CEU, CEU Universities, Madrid, Spain; ^2^Departamento de Farmacología, Facultad de Farmacia, Instituto Pluridisciplinar, Universidad Complutense de Madrid, Madrid, Spain; ^3^Departamento de Fisiología, Facultad de Medicina, Universidad Autónoma de Madrid, Madrid, Spain; ^4^Servicio de Endocrinología y Nutrición, Facultad de Medicina, Hospital Clínico San Carlos, Instituto de Investigaciones Sanitarias San Carlos, Universidad Complutense de Madrid, Madrid, Spain; ^5^Servicio de Cirugía, Facultad de Medicina, Hospital Clínico San Carlos, Instituto de Investigaciones Sanitarias San Carlos, Universidad Complutense de Madrid, Madrid, Spain; ^6^Departamento de Bioquímica, Facultad de Farmacia, Universidad Complutense de Madrid, Madrid, Spain

**Keywords:** obesity, bariatric surgery, matrix metalloproteinase-9, AMP-activated protein kinase, lactate dehydrogenase, intrinsic arterial stiffness

## Abstract

Bariatric surgery (BS) results in sustained weight loss and may reverse inflammation, metabolic alterations, extracellular matrix remodeling and arterial stiffness. We hypothesize that increased stiffening in omental arteries from obese patients might be associated with an increase in MMP activity and a decrease in p-AMPK, together with systemic oxidative stress and inflammation. Moreover, BS could contribute to reversing these alterations. This study was conducted with 38 patients of Caucasian origin: 31 adult patients with morbid obesity (9 men and 22 women; mean age 46 years and BMI = 42.7 ± 1.0 kg/m^2^) and 7 non-obese subjects (7 women; mean age 45 years and BMI = 22.7 ± 0.6 kg/m^2^). Seventeen obese patients were studied before and 12 months after BS. The stiffness index β, an index of intrinsic arterial stiffness, was determined in omental arteries and was significantly higher in obese patients. Levels of phosphorylated AMPK (p-AMPK^Thr-172^) and SIRT-1 were significantly lower in peripheral blood mononuclear cells (PBMCs) from obese patients than those from non-obese patients (*p* < 0.05) and were normalized after BS. Total and active MMP-9 activities, LDH, protein carbonyls and uric acid were higher in obese patients and reduced by BS. Moreover, there was a correlation between plasmatic LDH levels and the stiffness index β. BS has a beneficial effect on abnormal MMP-9, LDH and AMPK activities that might be associated with the development of arterial stiffness in obese patients. Since these parameters are easily measured in blood samples, they could constitute potential biomarkers of cardiovascular risk in morbid obesity.

## Introduction

Obesity constitutes a major social problem worldwide and is associated with insulin resistance, diabetes, and a higher risk of cardiovascular disease (CVD) (Morris, 2008). The increased accumulation of adipose tissue, especially in a visceral location, is strongly associated with a systemic pro-inflammatory and pro-oxidative state, leading to endothelial dysfunction, vascular remodeling, and arterial stiffness (Sutton-Tyrrell et al., 2001; Brunner et al., 2015; Morigami et al., 2016). Systemic arterial stiffness, assessed by pulse wave velocity (PWV) or the augmentation index is a classic feature of vascular aging and an independent predictor of cardiovascular morbidity and mortality (Turin et al., 2010; Vlachopoulos et al., 2010). Body fat and central adiposity have been suggested as predictors of accelerated arterial stiffness that develops at younger ages in obese patients (Nemes et al., 2008; Grassi and Diez, 2009; Kangas et al., 2013; Brunner et al., 2015; Satoh-Asahara et al., 2015). Stiffness index β, obtained from the stress–strain relationship (Dobrin, 1978), is currently a well-accepted marker of intrinsic arterial stiffness and it has been determined in several studies performed in human resistance arteries (Savoia et al., 2008; Fonck et al., 2009; Grassi et al., 2010). In addition, the increase of intrinsic stiffness of large arteries is now considered an independent predictor of cardiovascular (CV) diseases (Bouissou et al., 2014) and precedes changes in systemic arterial stiffness (Sehgel et al., 2013).

A variety of pathophysiological mechanisms might underlie the arterial stiffness development associated with obesity. AMP-activated protein kinase (AMPK) is a Ser/Thr protein kinase that senses cell energy status and contributes to restoring energy homeostasis (Hardie, 2007; Hue and Rider, 2007). AMPKα, the catalytic subunit of AMPK that is activated via phosphorylation (Dammanahalli and Sun, 2008; Chen et al., 2015), plays a protective role in vascular function (Garcia-Prieto et al., 2015b,c) and prevents the activation of mechanisms implicated in the development of arterial stiffness (for review see Garcia-Prieto et al., 2015a; Salt and Hardie, 2017). Dysregulation of AMPK is a pathogenic factor for the development of vascular disease (Lee et al., 2005) in genetic obese animal models (Ruderman et al., 2013) and humans (Gauthier et al., 2011; Ruderman et al., 2013). In severely obese patients before bariatric surgery (BS), the downregulation in AMPK activity in adipose tissue is linked to increased visceral adiposity, oxidative stress and inflammation (Gauthier et al., 2011; Xu et al., 2012).

Sirtuins, SIRT1–SIRT7, comprise a family of NAD^+^-dependent enzymes. The most studied is SIRT1, an NAD^+^-dependent protein deacetylase that protects against CVD (for review see Winnik et al., 2015). It has been shown to reduce arterial stiffness and endothelial dysfunction by inhibiting inflammatory and oxidant pathways (Gano et al., 2014; Fry et al., 2016). SIRT1 might be activated by caloric restriction and increased energy expenditure (Cohen et al., 2004; Civitarese et al., 2007; Chen et al., 2008), but downregulated by energy oversupply (Pfluger et al., 2008). In fact, SIRT1 expression is significantly reduced in adipose tissue from obese humans (Xu et al., 2013).

Matrix metalloproteinases (MMPs) are a large family of endopeptidases that can proteolyze all components of the extracellular matrix (ECM). MMP-2 and MMP-9 are involved in the cleavage of denatured collagen (gelatin), elastin and type IV collagen in blood vessels (Spinale, 2007) and in adipogenesis, vascular remodeling, and inflammation (Jaiswal et al., 2011). Therefore, MMP-9 seems to favor arterial stiffness and the development and progression of hypertension (Flamant et al., 2007; Zhou et al., 2007). In obese subjects, both MMP-2 and MMP-9 plasma levels are increased (Derosa et al., 2008; Andrade et al., 2012).

Weight loss is associated with an improvement in cardiometabolic disorders (Sjostrom et al., 2012). Since BS constitutes the most effective strategy for treating morbidly obese patients (Fruhbeck, 2015), it is a useful approach to search for new potential biomarkers of obesity-associated cardiovascular risk. We hypothesize that obese patients show a decrease in p-AMPK and an increase in MMPs activity associated with systemic oxidative stress and inflammation, and that body weight reduction derived from BS could significantly contribute to reversing these alterations. Therefore, the aim of this study was to evaluate the effect of BS on: (i) oxidative and inflammatory markers, (ii) AMPK activity and SIRT-1 expression, (iii) MMP-9 activity and LDH levels, and (iv) the stiffness index β. Changes in these parameters might allow the definition of potential biomarkers for the development of obesity-related arterial stiffness.

## Materials and Methods

### Study Population

**FIGURE 1:**
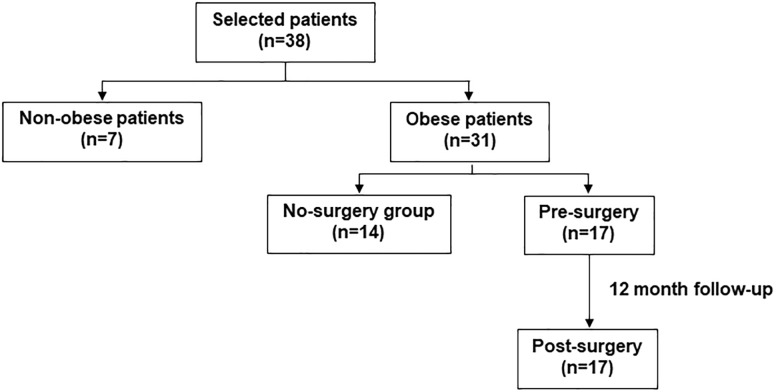
Scheme explaining the study design and the patient selection process.

As shown in Figure 1, this study was conducted with 38 subjects of Caucasian origin: 31 adult patients with morbid obesity [9 men and 22 women; mean age 46 years (range 27–62 years) and BMI = 42.7 ± 1.0 kg/m^2^] and 7 non-obese control subjects [7 women; mean age 45 years (range 33–55 years) and BMI = 22.7 ± 0.6 kg/m^2^ without other comorbidities]. The obese group was divided into two subgroups: (i) a group not undergoing BS (non-surgery group, 14 patients) and (ii) a group undergoing BS (pre-surgery group, 17 patients). The pre-surgery group was studied before and 12 months after BS. All pre-surgery patients underwent bariatric surgery [BS, elective laparoscopic bypass (53%) or sleeve gastrectomy (47%)] in the same center. The main inclusion criteria for BS was the presentation of more severe comorbidities (poorly controlled diabetes, ischemic heart disease, renal insufficiency or muscular neurological diseases) than in the non-surgery group. Patients were submitted to BS in chronologic order. Patients in the non-surgery group did not undergo BS because they rejected it for personal reasons and they preferred to receive counseling on lifestyle modifications (hypocaloric diet and exercise). They did not receive any pharmacological treatment for losing weight. Exclusion criteria were any past or present history of benign or malignant tumor, an inflammatory or autoimmune disorder and acute infections. Blood samples were obtained before BS and at 12 months follow-up. Information regarding anthropometric, clinical data and laboratory parameters was collected and included: age, body weight (BW), body mass index (BMI), waist circumference (WC), systolic (SBP), and diastolic blood pressure (DBP), complete hemogram, lipid profile, fasting glucose, glycosylated hemoglobin (HbA1c), insulin, homeostasis model assessment of insulin resistance index (HOMA-IR), C-reactive protein (CRP), lactate dehydrogenase (LDH), carbonyls, and uric acid (UA) levels. Small samples of omental adipose tissue were obtained from patients who underwent BS during the intervention. In addition, detailed clinical information was collected, including the patients’ comorbidities and medication. This study was approved by the Ethics Committee of the Hospital Clínico San Carlos and Universidad San Pablo-CEU and was conducted in compliance with the Helsinki Declaration. All patients signed a written informed consent form before they were included in the study.

### Blood Sample Collection

Blood samples from obese and non-obese subjects were collected under fasting conditions in citrated-BD Vacutainer Plus plastic serum tubes (Plymouth, United Kingdom). A total of 1 ml of blood was centrifuged at 3000 rpm for 10 min at 4°C to obtain plasma. A total of 5 ml of blood were diluted with PBS (pH = 7.4) (1:1 ratio) and submitted to a Ficoll-Hypaque (Sigma-Aldrich, United States) density gradient. After centrifugation at 1800 rpm for 30 min at 4°C, the mononuclear cell interface was collected, washed with PBS and centrifuged at 1200/1500 rpm for 5 min at 4°C. Both plasma and peripheral blood mononuclear cells (PBMCs) were frozen at -80°C until assay.

### Western Blot Analysis

Protein expression was determined in PBMCs by Western blot, as previously described (Somoza et al., 2007). Briefly, 20 μg protein samples were separated by SDS-PAGE gels. Primary antibodies anti p-AMPKα (Thr^172^) and AMPKα (1:1000 final dilution; Cell Signaling Biotechnology, Germany) and SIRT-1 (D1D7) (1:1000 final dilution; Cell Signaling Technology, United States) were applied overnight at 4°C. After washing, appropriate secondary antibodies (anti-rabbit or anti-mouse IgG-peroxidase conjugated) were applied for 1 h (1:5000 final dilution). Blots were washed, incubated in commercial enhanced chemiluminescence reagents (ECL Prime, Amersham Bioscience, United Kingdom) and bands were analyzed with the ChemiDoc XRS+ Imaging System (Bio-Rad, United States). To prove equal loading of samples, blots were incubated with β-actin antibody (1:5000 final dilution; Sigma-Aldrich, United States). Blots were quantified using Image Lab 3.0 software (Bio-Rad, United States). Expression values of p-AMPKα and SIRT-1 were normalized with AMPKα and β-actin, respectively.

### MMP-2 and MMP-9 Activity in Plasma

MMP-2 and MMP-9 gelatinase activity was determined by gelatin zymography. Total protein concentration was measured with the Bradford method (Bradford, 1976). Human plasma samples were diluted 1:1 with sample buffer and subjected to SDS-polyacrylamide gel electrophoresis (PAGE) containing 0.1% gelatin. Subsequently, gels were renatured and incubated for 12 h at 37°C. Gels were stained with Coomassie blue (Bio-Rad, United States) and, after destaining, gelatinolytic activity of MMP-2 and MMP-9 was detected as transparent bands against the background of the blue-stained gels. Transparent bands are the result of the digestion of gelatin, the substrate incorporated in polyacrylamide gel, because of enzymatic activity of MMP-2 and MMP-9. Bands were quantified using Image Lab 3.0 software (Bio-Rad, United States). The active MMP-9 form was determined using a commercial kit and following the manufacturer’s specifications (QuickZyme Biosciences, Netherlands).

### Plasma Carbonyls Analysis

Circulating carbonyl levels, as an index of oxidative marker on proteins, were assessed in plasma samples using the 2, 4-dinitrophenylhydrazine (DNPH)-based assay adapted to a microplate reader (Hawkins et al., 2009; Ruiz-Hurtado et al., 2014). Total protein carbonyls concentration was measured using the extinction coefficient of DNPH (ε = 22,000/mol/cm) and expressed as nmol/mg protein.

### Study of Vascular Mechanical Properties

Omental arteries were dissected from samples of omental adipose tissue from patients undergoing BS and were carefully cleaned of their surrounding adipose tissue. Mechanical properties were analyzed with a pressure myograph (Model P100, Danish Myo-Tech), as previously described (Briones et al., 2003; Gil-Ortega et al., 2016). Briefly, intraluminal pressure was set for equilibration at 70 mmHg for 30 min at 37°C in calcium-free KH (0Ca^2+^; omitting calcium and adding 10 mM EGTA) and bubbled with carbogen (95% O_2_/5% CO_2_). Afterwards, intraluminal pressure was augmented at 20 mmHg intervals (5, 20, 40, 60, 80, 100, 120, and 140 mmHg) and both external and internal diameters (D_i0Ca_, D_e0Ca_) were recorded and analyzed with Myoview software. From the D_e0Ca_ and D_i0Ca_ values, we calculated: (i) the circumferential wall strain (ε) = (D_i0Ca_ - D_00Ca_)/D_00Ca_, where D_00Ca_ is the diameter at 3 mmHg and D_i0Ca_ is the observed internal diameter for a given intravascular pressure in 0Ca^2+^ KH and (ii) the circumferential wall stress (β = (P × D_i0Ca_)/(2WT)), where P is the intraluminal pressure (1 mmHg = 1.334 dynes cm^-2^) (Briones et al., 2003). The stiffness index β, obtained from the stress-strain relationship, is proportional to Young’s incremental elastic modulus and is used to assess the intrinsic arterial stiffness independently of vessel geometry (Dobrin, 1978).

### Statistical Analysis

Continuous variables were compared using the Student’s *t*-test or one-way analysis of variance (ANOVA) following Newman–Keuls test. Data are reported as mean ± SEM. Correlation analyses were performed through linear regression and analyzed by Pearson’s correlation. All statistical analyses were performed using Graph-Pad Prism 7.0 or Origin 9.0 software. Statistical significance was set at *P* < 0.05.

## Results

### Clinical Characteristics

Anthropometric and clinical parameters at baseline and at 12 months follow-up are summarized in Table 1. At baseline, the obese group, especially the pre-surgery group, exhibited a higher number of CV risk factors than non-obese subjects, who did not present any comorbidity. In the pre-surgery group, 14 (82.4%) patients were diagnosed with type 2 diabetes mellitus (T2DM, 76.5% treated with metformin therapy), 10 (58.8%) with hypertension (all treated with either an ACEi or ARB) and 11 (64.7%) with dyslipidemia (all treated with statins). In the non-surgery group, no patient was diagnosed with T2DM, 8 (57.1%) patients were diagnosed with hypertension (all treated with ACEi or ARB) and 9 (64.3%) with dyslipidemia (all treated with statins).

**Table 1:** Patients’ characteristics.

Variable	Non-obese control (*N* = 7)	Obese (*N* = 31)		Obese post-surgery (*N* = 17)
			
		No surgery	Pre-surgery	
		(*N* = 14)	(*N* = 17)	
Sex-N (%) female	7 (100%)	11 (78.6%)	11 (64.7%)	—
Age (years)	45.0 ± 4.5	41 ± 2.7	48.6 ± 2.5	—
Weight (Kg)	58.3 ± 3.3^$^	121.0 ± 6.3^###^	113.7 ± 4.0^###^	79.9 ± 4.0^∗∗∗^
BMI (kg/m^2^)	22.7 ± 0.6^$$^	43.7 ± 1.9^###^	41.8 ± 0.9^###^	28.6 ± 1.1^∗∗∗^
WC (cm)				
Men	—	150.0 ± 5.8	136.1 ± 5.6	101.0 ± 6.0^∗∗^
Women	83.6 ± 1.8	111.4 ± 4.6^###^	118.0 ± 3.2^###^	84.4 ± 2.9^∗∗∗^
Hypertension-N (%)	—	8 (57.1%)	10 (58.8%)	6 (35.3%)
SBP (mmHg)	127.7 ± 8.4	121.5 ± 2.4	129.8 ± 4.3	134.8 ± 4.6
DBP (mmHg)	83.4 ± 2.3	77.7 ± 2.2	77.9 ± 2.4	79.7 ± 3.3
ACEi/ARBs user	—	8 (57.1%)	10 (58.8%)	6 (35.3%)
Dyslipidemia-N (%)	—	9 (64.3 %)	11 (64.7 %)	5 (29.4 %)
LDL-cholesterol (mg/dL)	119.1 ± 6.1	116.3 ± 6.9	120.9 ± 18.9	101.2 ± 8.9
HDL-cholesterol (mg/dL)	61.9 ± 2.5	48.4 ± 2.5^#^	48.3 ± 3.5^#^	48.4 ± 3.1^#^
Total cholesterol (mg/dL)	194.3 ± 9.0	210.2 ± 15.7	185.0 ± 10.1	172.9 ± 11.6
Triglycerides (mg/dL)	65.6 ± 5.4^$^	142.1 ± 22.1^#^	171.2 ± 22.0^##^	126.6 ± 13.4
Statins user	—	9 (64.3%)	11 (64.7%)	5 (29.4%)
T2DM-N (%)	—	—	14 (82.4%)	8 (47.0%)
Glucose (mg/dL)	88.7 ± 2.0	104.4 ± 4.3	162.6 ± 15.7^###,&^	102.8 ± 6.5^+++^
HbA1c (mmol/mol)	—	38.0 ± 1.3	55.3 ± 4.4^&&^	41.9 ± 2.4^++^
Insulin (μIU/ml)	8.8 ± 0.2	33.2 ± 7.6^##^	36.2 ± 5.9^##^	11.1 ± 2.01^∗∗∗^
HOMA-IR	1.7 ± 0.3	8.4 ± 1.8^##^	6.9 ± 1.3^##^	2.7 ± 0.6^+++^
Metformin user	—	—	13 (76.5%)	8 (47.1%)


**Demographic, anthropometric, obesity-related comorbidities, and biochemical parameters. Data are expressed as mean ± SEM. Data were compared using one-way ANOVA, ^∗∗^P < 0.01, ^∗∗∗^P < 0.001 vs. obese; ^#^P < 0.05, ^##^P < 0.01, ^###^P < 0.001 vs. control; ^$^P < 0.05, ^$$^P < 0.01 vs. post-surgery; ^&^P < 0.05, ^&&^P < 0.01 vs. non-surgery; ^++^P < 0.01, ^+++^P < 0.001 vs. pre-surgery. Newman-Keuls test. BMI, Body mass index; WC, waist circumference; SPB, systolic; DBP, diastolic blood pressure; ACEi, angiotensin converting enzyme inhibitors; ARBs, angiotensin receptor blockers; LDL-cholesterol, low-density lipoprotein; HDL, high-density lipoprotein-cholesterol; T2DM, type 2 diabetes mellitus; HbA1c, glycosylated hemoglobin; HOMA-IR, homeostasis model assessment of insulin resistance index.**

Anthropometric parameters including BW, BMI and WC were significantly higher in obese patients than in controls (*P* < 0.001, Table 1) and significantly reduced 12 months after BS (% of reduction: BW = 29.7%; BMI = 31.6%; and WC = 28.5% in female and 25.8% in male) compared with their corresponding pre-surgery values (*P* < 0.001 in all cases). BS significantly reduced fasting glucose level and improved insulin sensitivity (HOMA-IR) (*P* < 0.001). Lower HDL-cholesterol and higher triglyceride (TG) levels were detected in obese patients than in the control group. However, although these parameters were not significantly modified by BS, a reduction in TG levels was observed (*P* = 0.068 vs. obese). SBP and DBP were similar between groups, which shows proper blood pressure control in obese patients treated with ACEi/ARBs. In addition, as shown in Table 1, the intake of antihypertensive drugs was reduced after BS.

Since no differences were detected between non-surgery and pre-surgery groups in carbonyls, UA, LDH, inflammatory markers, AMPK and MMP-9 activities and SIRT-1 levels (data not shown), these parameters were analyzed altogether, and we will refer to them as the obese group.

### Bariatric Surgery Significantly Reduces Protein Oxidative Damage and Inflammatory Markers/Cells in Obese Patients

**FIGURE 2:**
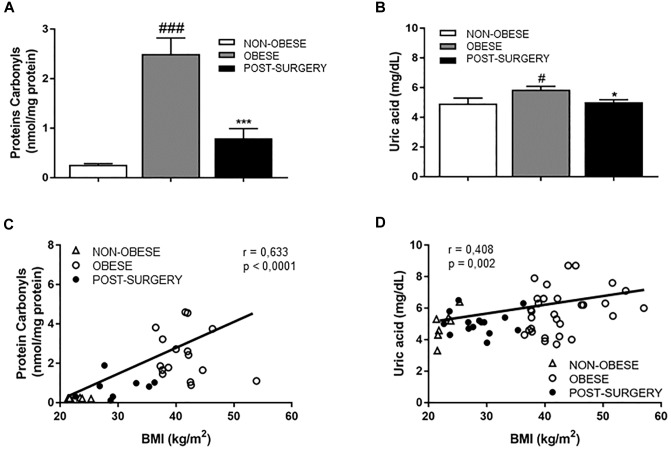
**(A,B)** Diagram bars shows protein carbonyls **(A)** and uric acid (UA) concentrations **(B)** in plasma samples from non-obese (white bar, *n* = 7), obese (gray bar, *n* = 31) and post-surgery (black bar, *n* = 17) patients. **(C)** Correlation between protein carbonyl concentrations in plasma and BMI. **(D)** Correlation between plasmatic UA levels and BMI. White triangles correspond to non-obese, white circles correspond to obese and black circles correspond to Post-surgery. Data are expressed as mean ± SEM. ^#^*p* < 0.05 and ^###^*p* < 0.001 compared with the non-obese group. ^∗^*p* < 0.05 and ^∗∗∗^*p* < 0.001 compared with the obese group.

Levels of plasma carbonyls (Figure 2A; *P* < 0.001) and UA (Figure 2B; *P* < 0.05), were significantly higher in obese patients than in controls. After BS, carbonyls and UA levels were significantly reduced (*P* < 0.05 vs. obese) and correlated positively with BMI (*r* = 0.633 and *P* < 0.001; Figure 2C and *r* = 0.408 and *P* < 0.01; Figure 2D, respectively).

**FIGURE 3:**
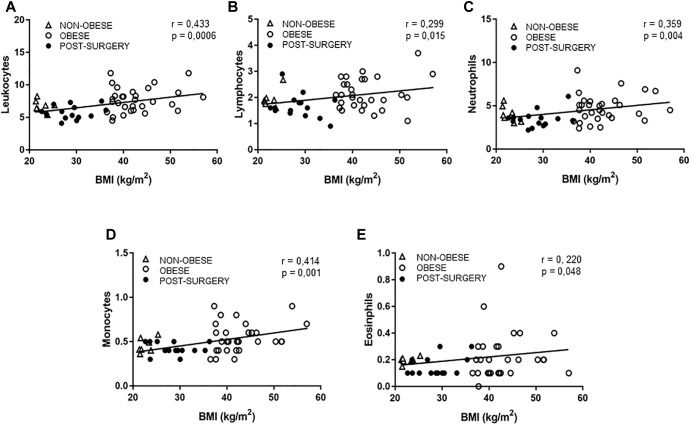
**(A–E)** Correlations between BMI and total leukocytes **(A)**, lymphocytes **(B)**, neutrophils **(C)**, monocytes **(D),** and eosinophils **(E)**. White triangles correspond to non-obese, white circles correspond to obese and black circles correspond to Post-surgery.

As shown in Table 2, CRP was over sixfold higher in obese patients than in the non-obese group (*P* < 0.01), but significantly reduced after BS (*P* < 0.05). We also quantified in blood samples the cell number of neutrophils, eosinophils, monocytes and lymphocytes. Immune cell number was similar in obese and control subjects. Post-surgery patients exhibited a significant reduction in the amount of these immune cells, except for eosinophils (*P* < 0.05 vs. obese; Table 2). Simple regression analysis revealed a positive correlation between total leukocytes, lymphocytes, neutrophils, monocytes or eosinophils and BMI (Figure 3).

**Table 2:** Hemogram and CRP levels.

Variable	Non-obese control (*N* = 7)	Obese (*N* = 31)		Obese post-surgery (*N* = 17)
			
		No surgery (*N* = 14)	Pre-surgery (*N* = 17)	
**HEMOGRAM**		
Leukocytes (^∗^10^3^μl)	6.8 ± 0.3	7.8 ± 0.5	7.7 ± 0.5	5.7 ± 0.2^∗∗∗^
Neutrophils (^∗^10^3^ μl)	4.1 ± 0.4	4.9 ± 0.3	4.6 ± 0.4	3.4 ± 0.2^∗∗^
Eosinophils (^∗^10^3^ μl)	0.2 ± 0.09	0.22 ± 0.03	0.25 ± 0.05	0.16 ± 0.02
Monocytes (^∗^10^3^ μl)	0.5 ± 0.03	0.52 ± 0.03	0.56 ± 0.05	0.42 ± 0.18^∗∗^
Lymphocytes (^∗^10^3^ μl)	1.9 ± 0.1	2.2 ± 0.2	2.2 ± 0.1	1.7 ± 0.1^∗∗^
CRP (mg/dl)	0.16 ± 0.01	1.03 ± 0.2^##^	1.39 ± 0.31^##^	0.47 ± 0.09^∗^


**Data are expressed as mean ± SEM. Data were compared using one-way ANOVA, ^∗^P < 0.05, ^∗∗^P < 0.01, ^∗∗∗^P < 0.001 vs. obese, ^##^P < 0.01 vs. control. Newman–Keuls test.**

### Bariatric Surgery Improves Both AMPK Activity and SIRT-1 Expression in PBMCs From Obese Patients

**FIGURE 4:**
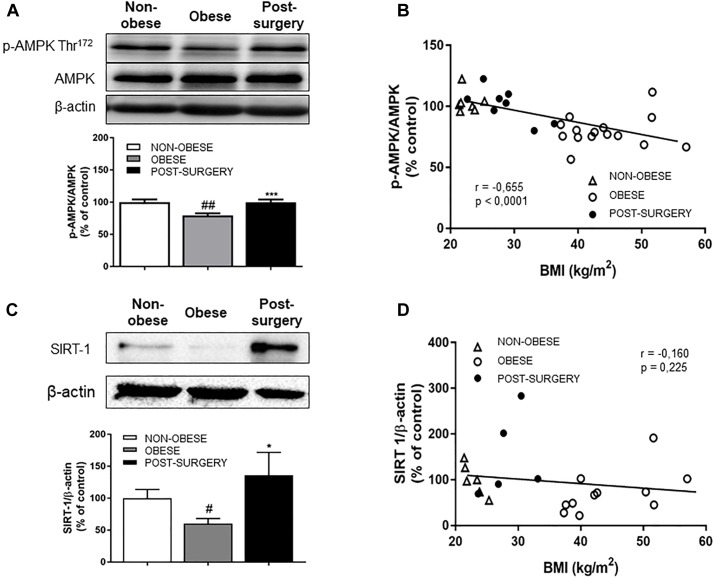
**(A)** Representative immunoblots of p-AMPK/AMPK and β-actin expression in PBMCs from non-obese, obese and post-surgery patients. Diagram bars show the result of densitometric analysis of p-AMPK/AMPK immunoblots expressed as a percentage of p-AMPK/AMPK in the non-obese group. **(B)** Correlation between p-AMPK/AMPK and BMI. **(C)** Representative immunoblots of SIRT1 and β-actin expression in PBMCs from non-obese, obese and post-surgery patients. Diagram bars show the result of densitometric analysis of SIRT1/β-actin immunoblots expressed as a percentage of SIRT1/β-actin in the non-obese group. **(D)** Correlation between SIRT1/β-actin and BMI. Data are expressed as mean ± SEM of ≥ 7 determinations per group. ^#^*p* < 0.05 and ^##^*p* < 0.01 compared with the non-obese group. ^∗^*p* < 0.05 and ^∗∗∗^*p* < 0.001 compared with the obese group.

p-AMPKα^Thr172^ levels (Figure 4A) were significantly lower in PBMCs from obese patients than in those from the control group (*P* < 0.01) but were restored to normal values 12 months after surgery (*P* < 0.001 vs. obese levels). The negative correlation between p-AMPKα^Thr172^ and BMI (Figure 4B; *r* = 0.655, *P* < 0.001) evidences greater AMPK activity in post-surgery patients with a lower BMI than in obese patients with a higher BMI. A negative correlation was observed between p-AMPKα^Thr172^ and plasma carbonyls (*r* = -0.709 and *P* < 0.001).

SIRT-1 expression was significantly lower in obese patients (*P* < 0.05 compared with controls) but reached normal values after BS (*P* < 0.05; Figure 4C). There was no correlation between SIRT-1 expression and BMI (Figure 4D; *r* = -0.16, *P* = 0.225).

### Bariatric Surgery Significantly Reduces MMP-9 Activity and Lactate Dehydrogenase Levels in Plasma From Obese Patients

**FIGURE 5:**
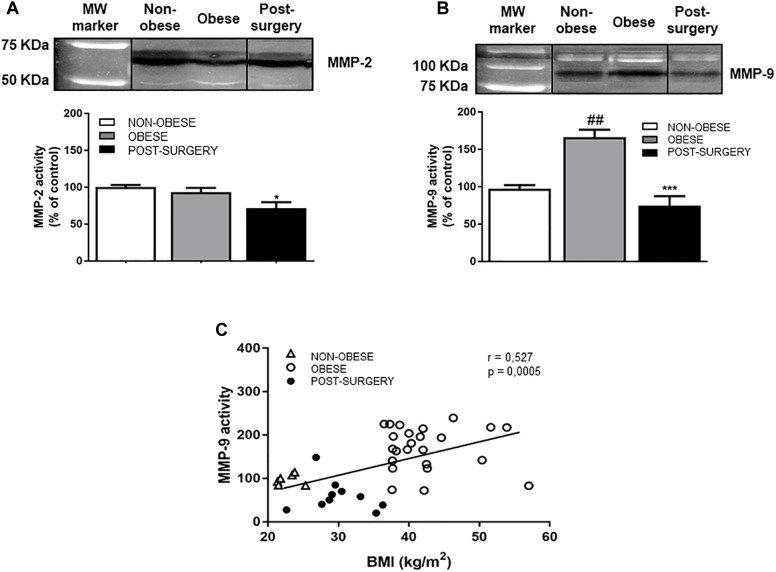
**(A)** Representative cropped immunoblots of MMP-2 activity in plasma samples from non-obese, obese and Post-surgery patients. Diagram bars show the result of densitometric analysis of MMP-2 activity expressed as a percentage of MMP-2 activity in the non-obese group. **(B)** Representative cropped immunoblots of MMP-9 activity in plasma samples from non-obese, obese and Post-surgery patients. Diagram bars show the result of densitometric analysis of MMP-9 activity expressed as a percentage of MMP-9 activity in the non-obese group. **(C)** Correlation between MMP-9 activity and BMI. White triangles correspond to non-obese, white circles correspond to obese and black circles correspond to Post-surgery. Data are expressed as mean ± SEM of ≥ 7 determinations per group. ^##^*p* < 0.01 compared with the non-obese group. ^∗^*p* < 0.05 and ^∗∗∗^*p* < 0.001 compared with the obese group.

Gelatinase MMP-2 activity was similar in obese and non-obese subjects, but significantly lower after BS (Figure 5A, *P* < 0.05 vs. obese). Gelatinase MMP-9 activity was significantly higher in obese patients than in controls (Figure 5B; *P* < 0.01) and normalized after BS (*P* < 0.001 vs. obese). Likewise, specific measurement of the active MMP-9 form by ELISA revealed a significant reduction in active MMP-9 levels after BS (obese = 0.205 ± 0.01; Post-surgery = 0.153 ± 0.02; *P* < 0.01), which positively correlated with BMI (Figure 5C; *r* = 0.527, *P* < 0.001).

There was also a positive correlation between plasmatic MMP-9 activity and protein carbonyls (*r* = 0.630, *P* < 0.001), and with leukocyte and neutrophil number (*r* = 0.300 and *P* < 0.05 and *r* = 0.298 and *P* < 0.05, respectively).

**FIGURE 6:**
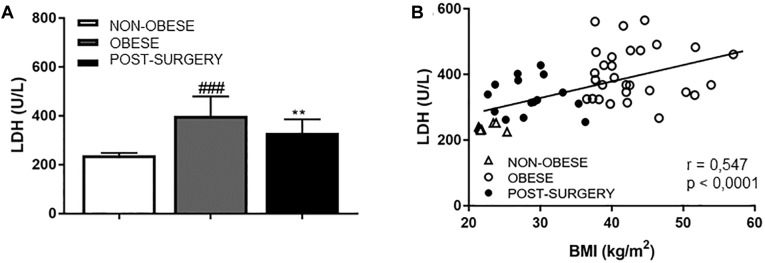
**(A)** Diagram bars shows LDH concentrations in plasma samples from non-obese, obese and Post-surgery patients. Data are expressed as mean ± SEM of ≥7 determinations per group. ^###^*p* < 0.001 compared with the non-obese group. ^∗∗^*p* < 0.01 compared with the obese group. **(B)** Correlation between LDH concentrations and BMI. White triangles correspond to non-obese, white circles correspond to obese and black circles correspond to Post-surgery.

Lactate dehydrogenase (LDH) was significantly higher in obese patients than in controls (*P* < 0.001) and significantly diminished in the Post-surgery group (Figure 6A, *P* < 0.01 vs. obese). A positive correlation was observed between LDH levels and BMI (Figure 6B; *r* = 0.547, *P* < 0.001).

Mechanical studies performed in omental arteries from obese patients evidenced a stiffness index β of 13.9 ± 2.5 (*n* = 11), as an index of intrinsic arterial stiffness. In obese patients, we found a significant correlation between LDH and the stiffness index β (*r* = 0.829, *P* < 0.001).

## Discussion

The study demonstrates that both abnormal plasma MMP-9 and PBMCs AMPK activities are reversed by BS. Interestingly, we found a positive correlation between leukocyte and neutrophil number and MMP-9 activity. Moreover, we describe for the first time a relationship between plasmatic LDH levels and the stiffness index β, as a marker of intrinsic arterial stiffness in omental arteries from morbid obese patients, and a correlation between AMPK and MMP-9 activity and LDH values. Finally, as BS has been shown to effectively normalize all these parameters, we suggest that plasma MMP-9 and PBMCs AMPK activities might potentially be used as markers of cardiovascular risk in patients with morbid obesity.

Obesity is an established risk factor for several CVDs and it may be associated with early vascular alterations. The presence of classic CV risk factors (T2DM, hyperlipidemia and hypertension) in obese subjects increases the risk of accelerating arterial stiffness, especially if obesity appears at early ages (Wildman et al., 2003; Woo et al., 2004; Sato et al., 2005; Safar et al., 2006). In our study, the mean age of obese subjects was 46 years and over 70% of them already presented CV risk factors. Insulin resistance and a low-grade inflammatory state are common physiopathological mechanisms in obesity and T2DM. Therefore, we did not detect differences in arterial stiffness-related parameters between obese groups (non-surgery vs. pre-surgery). Moreover, 11 of 17 patients already exhibited an elevated intrinsic stiffness in omental arteries, as shown by stiffness index β over 10.2, which indicates pathological stiffness in this group (Sato et al., 2013) and is a predictor of all-cause mortality (Willum-Hansen et al., 2006; Mitchell et al., 2010; Vlachopoulos et al., 2010). Previous studies also evidenced increased arterial stiffness in obese patients (Dangardt et al., 2013; Pal and Radavelli-Bagatini, 2013; Arner et al., 2015; Cote et al., 2015; Strasser et al., 2015; Manco et al., 2017) assessed by the determination of either the PWV or the augmentation index; parameters that are dependent on blood pressure levels and markers of systemic but not intrinsic arterial stiffness (Matsui et al., 2004). In addition, Savoia et al. (2008), have described an increased stiffness index β in gluteal subcutaneous arteries from hypertensive patients compared to normotensive subjects, thus suggesting a relationship between an increased stiffness and BP in hypertension. Moreover, a recent study from our group performed in an animal model of diet-induced obesity has also shown an association between the stiffness index β in mesenteric resistance arteries and PWV (Gil-Ortega et al., 2016). Nevertheless, a relationship between intrinsic stiffness of omental arteries stated by the stiffness index β and PWV has not been clearly stated yet. Nevertheless, though no direct comparison can be established with our results, changes in intrinsic arterial stiffness precede changes in systemic arterial stiffness (Sehgel et al., 2013). The early identification of patients with altered intrinsic stiffness might thus be key for risk assessment and the prevention of further CV damage.

Several pathophysiological mechanisms like chronic low-grade inflammation and immune activation, oxidative stress, and ECM remodeling are involved in the interaction between obesity and arterial stiffness (Leopold, 2013; Aroor et al., 2018). Moreover, a strong association has been described in women between obesity and increased total lymphocyte counts and white blood cells (Womack et al., 2007). Our obese patients had very high CRP levels, but reached normal levels after BS. CRP directly induces endothelial dysfunction through a reduction in nitric oxide production (Venugopal et al., 2002) and favors oxidative stress. This is in accordance with the oxidative protein damage and increased UA levels observed in the obese group. UA is considered a powerful scavenger of free radicals, which are increased in patients with obesity and insulin resistance, probably as a compensatory mechanism to protect against oxidative damage (Fabbrini et al., 2014). The fact that these parameters reach normal values is in accordance with the beneficial effect of BS on inflammatory and oxidative stress status (Kopp et al., 2005; Sheu et al., 2008). Moreover, there is a reduction in lymphocytes, leukocytes, neutrophils and monocytes in the hemogram of obese patients after BS that correlates with BMI.

Inflammation may induce elastin degradation and changes in the ECM composition through MMP (MMP-2 and MMP-9) activation, thus resulting in an accumulation of stiffer, uncoiled collagen (Galis and Khatri, 2002). Similarly to other studies, our obese patients show a significant increase in MMP-9 activity (Laimer et al., 2005; Derosa et al., 2008; Madsen et al., 2009) that correlates with BMI. Under the influence of pro-inflammatory cytokines, neutrophils induce MMP-9 activity and contribute to microvascular complications (Galis and Khatri, 2002). In this context, we observed a positive, significant relationship in obese patients between neutrophil number and MMP-9 activity, which suggests that neutrophil number may be highlighted as a marker of ECM remodeling. Moreover, the positive correlation between MMP-9 activity and circulating carbonyl levels in obese patients is consistent with the traditional concept that MMP-9 expression is inducible through several mechanisms, the most important of which is the action of reactive oxygen species (ROS) (Kuhad et al., 2015). Pleiotropic effects of statins include the reduction of MMP-9 activity and other inflammation markers (Guerrero-Romero and Rodriguez-Moran, 2005). Therefore, an effect of the treatment on these parameters in addition to BS cannot be excluded.

One of the most interesting results of our study is the increased LDH level in obese patients that is reduced after BS and correlates with BMI. Nutritional diseases such as obesity have been associated with changes in mitochondrial oxidative phosphorylation and changes in the phenotype of vascular smooth muscle cells (VSMCs) (Chiong et al., 2014). The correlation between LDH and the stiffness index β might suggest an association between LDH and arterial stiffness associated with obesity.

Lymphocyte mitochondrial physiology seems to be associated with nutritional status (Briet et al., 2003) and other cell type metabolic alterations. Therefore, it might be considered an interesting biomarker of metabolic status (Cortez et al., 2012). In this context, we used PBMCs to determine both AMPK activity and SIRT-1 expression in controls vs. obese vs. Post-surgery patients. AMPK is a metabolic sensor. It reduces inflammation, oxidative stress and vascular remodeling (Garcia-Prieto et al., 2015a) and is dysregulated in obesity (Gauthier et al., 2011; Xu et al., 2012). In accordance with these studies, we detected a significant reduction in AMPK activity in obese patients that was reversed by BS. Notably, AMPK activation also favors the switch of the synthetic phenotype of VSMCs toward a contractile phenotype (Stone et al., 2013). SIRT-1 can activate AMPK but also has wide-ranging effects on metabolic homeostasis, mainly as a regulator of mitochondrial integrity and by reducing inflammatory and oxidative stress tone (Kida and Goligorsky, 2016). In our study, we found an increase in SIRT-1 expression after BS. Moreover, since it has been suggested that SIRT-1 plays a key role in CV protection, SIRT-1 overexpression could contribute to reducing CV risk after BS.

Altogether, these data demonstrate that alterations in both MMP-9 and AMPK activities might play a key role in the development of arterial stiffness. In addition, other factors that are altered in obesity like LDH, SIRT-1 expression, oxidative stress markers or immune disorders could also contribute to vascular damage.

## Conclusion

A limitation of this study is that we did not measure intrinsic arterial stiffness in omental arteries after BS for obvious ethical reasons. Thus, we cannot conclude that modification in abnormal AMPK, LDH, and MMP-9 activities has an impact on intrinsic arterial stiffness in these patients. Further studies are required to answer this question. Nonetheless, several studies have demonstrated the relationship between weight loss (i.e., after lifestyle modifications or surgical interventions) and the reduction of arterial stiffness in both obese animal models and morbid obese patients (Shargorodsky et al., 2006; Rider et al., 2010; Weisbrod et al., 2013; Brunner et al., 2015; Hudson et al., 2017; Manco et al., 2017; Backdahl et al., 2018). In conclusion, since pAMPK, LDH and MMP-9 activities might be easily measured in blood samples, they could be systematically used as potential markers of CV risk and allow for the early identification of patients with an altered intrinsic stiffness and the prevention of further CV damage.

## Ethics Statement

This study was carried out in accordance with the recommendations of the Ethics Committee of the Hospital Clínico San Carlos. The protocol was approved by the Ethics Committee of the Hospital Clínico San Carlos.

## Author Contributions

CG-P, MG-O, IA, MF-A, and BS participated in the design of the study, the analysis and interpretation of the results obtained, and the main manuscript writing. CG-P and MM-R performed Western blot and gelatin zymography analyses. MG-O performed active MMP-9 determination. DR-C participated in carbonyl determination. EV-M and MG-O performed mechanical studies in omental arteries. EB, AT, AS-P, and MR provided human samples, clinical data, and plasmatic parameters. MF-A, BS, IA, and MR ensured funding. All authors reviewed the manuscript.

## Conflict of Interest Statement

The authors declare that the research was conducted in the absence of any commercial or financial relationships that could be construed as a potential conflict of interest.

## Abbreviations

AMPK: AMP-dependent protein kinase
BMI: body mass index
BS: bariatric surgery
BW: body weight
CRP: C reactive protein
CVD: cardiovascular disease
DBP: diastolic blood pressure
ECM: extracellular matrix
HOMA-IR: insulin resistance index
LDH: lactate dehydrogenase
MMP: matrix metalloproteinases
ROS: reactive oxygen species
SBP: systolic blood pressure
SIRT: sirtuin
UA: uric acid
VSMCs: vascular smooth muscle cells
WC: waist circumference
